# Reduced audiovisual temporal sensitivity in Chinese children with dyslexia

**DOI:** 10.3389/fpsyg.2023.1126720

**Published:** 2023-04-20

**Authors:** Huiduo Wu, Haidan Lu, Qing Lin, Yuhong Zhang, Qiaoyun Liu

**Affiliations:** ^1^College of Child Development and Education, Zhejiang Normal University, Hangzhou, China; ^2^Faculty of Education, East China Normal University, Shanghai, China; ^3^Department of Preschool Education, China Women’s University, Beijing, China; ^4^The College of Education Science, Xinjiang Normal University, Urumqi, China

**Keywords:** audiovisual temporal sensitivity, simultaneity judgment, temporal order judgment, time binding window, dyslexia, cross-modal

## Abstract

**Background:**

Temporal processing deficits regarding audiovisual cross-modal stimuli could affect children’s speed and accuracy of decoding.

**Aim:**

To investigate the characteristics of audiovisual temporal sensitivity (ATS) in Chinese children, with and without developmental dyslexia and its impact on reading ability.

**Methods:**

The audiovisual simultaneity judgment and temporal order judgment tasks were performed to investigate the ATS of 106 Chinese children (53 with dyslexia) aged 8 to 12 and 37 adults without a history of dyslexia. The predictive effect of children’s audiovisual time binding window on their reading ability and the effects of extra cognitive processing in the temporal order judgment task on participants’ ATS were also investigated.

**Outcomes and results:**

With increasing inter-stimulus intervals, the percentage of synchronous responses in adults declined more rapidly than in children. Adults and typically developing children had significantly narrower time binding windows than children with dyslexia. The size of visual stimuli preceding auditory stimuli time binding window had a marginally significant predictive effect on children’s reading fluency. Compared with the simultaneity judgment task, the extra cognitive processing of the temporal order judgment task affected children’s ATS.

**Conclusion and implications:**

The ATS of 8–12-year-old Chinese children is immature. Chinese children with dyslexia have lower ATS than their peers.

## 1. Introduction

Audiovisual processing is indispensable for conventional reading acquisition. Efficient reading requires a reliable and automated audiovisual cross-modal connection between characters and phonology (Naples et al., 2009; Hahn et al., 2014). Developmental dyslexia involves an individual’s specific difficulties with reading; this problem is not caused by intellectual deficits, obvious sensory or neurodevelopmental disorders, motivation, or a lack of educational opportunities (Lyon et al., 2003). The decoding defect is one of the core deficits of dyslexia, and manifests as difficulty recognizing and spelling words accurately or fluently (Franzen et al., 2021). Birch and Belmont (1964) proposed that the occurrence of dyslexia may be due to defects in the integration of audiovisual processing. Audiovisual temporal sensitivity (ATS) refers to an individual’s ability to perceive the appearance time of visual and auditory stimuli (Francisco et al., 2017a). For example, people usually perceive visual and auditory stimuli that are very close in time as the same source. However, if the time interval between them is longer, they will be perceived as stimuli from different sources. ATS is an indicator reflecting audiovisual integration ability (Mégevand et al., 2013). Some researchers believe a deficit in ATS would lead to an expanded audiovisual time binding (or integration) window (TBW). It blurs the correspondence between characters and phonetic representations, which affects the speed and accuracy of decoding characters (Hairston et al., 2005; Hahn et al., 2014; Wallace and Stevenson, 2014).

### 1.1. The effects of experimental paradigms on ATS

The audiovisual temporal order judgment (TOJ) and audiovisual simultaneity judgment (SJ) tasks are the two most commonly used experimental paradigms for investigating an individual’s ATS (Francisco et al., 2017a). In TOJ tasks, participants are required to judge whether a visual or an auditory stimulus was presented first, while in SJ tasks, they are instructed to judge whether they perceive auditorily- and visually-presented stimuli as synchronous or asynchronous. In the two experimental paradigms, several indexes, such as the percentage of error response or synchronous perceptions, the size of the audiovisual TBW, and the point of subjective simultaneity (PSS, at which an individual is most likely to perceive the two inputs as synchronous) have often been used to reflect the individual’s ATS level (Wallace and Stevenson, 2014). However, researchers believe that the processing mechanisms differ between the two task paradigms. One view is that the two tasks measure completely different cognitive processes (Zampini et al., 2003; Vatakis et al., 2008). However, Jaśkowski (1991) proposed that there are some overlapping cognitive mechanisms between the two tasks. The TOJ task includes two cognitive processes: simultaneous and sequential processing. The SJ task only involves simultaneous processing. In recent years, some electrophysiological studies have found evidence for Jaśkowski’s view. In the TOJ task, participants had transient activations in several brain regions of the left hemisphere, but some of these activations were not found in the SJ task. There are overlapping but different neural mechanisms between the two tasks. Extra cognitive processing was performed in the TOJ task versus in the SJ task (Binder, 2015; Love et al., 2018), such as a judgment of “order” (Matsuzaki et al., 2014). Researchers have asserted that the SJ task was better than the TOJ task when the research aim was mainly to investigate the individual’s perception of audiovisual simultaneity (Van Eijk et al., 2008).

### 1.2. The development of ATS in children with or without dyslexia who were native speakers of the alphabetic language

Studies of children and adults with alphabetic language show that ATS requires a long time to mature. Infants needed a longer stimulus onset asynchrony time than adults when judging the asynchrony of auditory and visual stimuli (Lewkowicz, 1996). Throughout the entire pre-school (Lewkowicz and Flom, 2014) and school-age period (Hillock-Dunn and Wallace, 2012), the width of the audiovisual TBW in children continues to narrow; it will not narrow significantly until adolescence. However, the developmental characteristics of children’s ATS have not been consistently described across studies. Hillock et al. (2011) found that when auditory stimuli preceded visual stimuli (AV), children made more synchronous judgments than adults when the stimulus onset asynchrony time was between 150 and 350 ms. When visual stimuli preceded auditory stimuli (VA), there was no significant difference in the synchronous judgment rate between children and adults. However, Kaganovich (2016) demonstrated that children perceived asynchronous auditory and visual stimuli as synchronous more than adults, when the stimulus onset asynchrony time was longer than 100 ms, whether in the VA or AV. They also discovered different developmental trajectories of children’s ATS in VA and AV. The development of children’s temporal sensitivity in AV was slower than that in VA. Hillock et al. (2011) indicated that children’s audiovisual TBW was more symmetrical than that of adults. Compared with the children, adults’ PSS was clearly biased toward VA. However, the electrophysiological study conducted by Froyen et al. (2008, 2009) revealed that adults displayed the largest mismatch negativity amplitude when letters and phonology were presented simultaneously. Whereas, in fifth-grade children, enhanced mismatch negativity amplitude was observed only when the letters were presented 200 ms before the phonology. This implies that the PSS of adults is closer to the objective simultaneity point, while the PSS of children is biased toward VA.

Most studies of ATS in persons with dyslexia regarding alphabetic language have focused on adults. These studies show that ATS defects occur not only at the language level, but also at the general nonverbal cognitive level (Francisco et al., 2017a,b). The ATS impairment in participants with dyslexia, in relation to reading, worsened with age (Virsu et al., 2003). Laasonen et al. (2000) investigated the cross-modal temporal sensitivity of Finnish children with dyslexia (CD) aged 8 to 12. They found that the children have a higher judgment threshold of audiovisual simultaneity than their age-matched typically developing (TD) peers. However, the characteristics of ATS in VA and AV and its effect on reading were not explored in detail.

### 1.3. The effect of ATS on the reading ability of Chinese children

There are major differences between Chinese and alphabetic languages in terms of orthography and phonetic features, which may result in different requirements for visual and auditory processing in the two types of languages while decoding.

Liu et al. (2019) found that the response accuracy of Chinese CD in the audiovisual TOJ task was significantly lower than that of age-matched TD peers. The accuracy can independently predict their character recognition. However, the study followed Tallal and Piercy (1973) classic TOJ task paradigm, in which participants are not only asked to judge the sequence of stimuli, but also the characteristics of the stimuli (e.g., the shape of graphics and the level of sound frequencies; Liu et al., 2019). This significantly increased the cognitive load of the children. There may also have been confusion about temporal perception ability along with other cognitive functions, such as attention and working memory (Kaganovich et al., 2014). As a result, it was difficult to determine whether differences in ATS between dyslexic and TD children were due to differences in temporal perception or other cognitive skills. Chen et al. (2016) found that Chinese CD gain multisensory facilitation in a wider audiovisual stimulus onset asynchrony time in the temporal ventriloquism task, indicating that CD have a broader audiovisual TBW. However, the characteristics of the audiovisual TBW of CD have not been elaborated on.

Based on the above research, this study puts forward the following questions: (1) What are the differences in the ATS of Chinese children with or without dyslexia and adults? (2) What are the characteristics of the audiovisual TBW for children with or without dyslexia and adults? Does TBW have a predictive effect on children’s reading ability? (3) Does the extra cognitive processing in the TOJ task significantly affect children’s ATS and reading ability compared with the SJ task?

The first purpose was to describe the characteristics of ATS and TBW in Chinese children with or without dyslexia, and in adults. The second was to investigate the predictive effect of the size of audiovisual TBW on children’s reading ability. The third aim involved examining the effect of extra cognitive processing during the TOJ task on children’s ATS and reading ability, to clarify the reliability of this paradigm in studying the effect of ATS on children’s reading skills.

This study hypothesized that (1) the ATS of CD would lag behind that of age-matched TD children. The ATS of Chinese children aged 8 to 12 has not matured and reached the adult level. (2) The size of audiovisual TBW in CD is larger than that of TD children. The size of audiovisual TBW has a significant predictive effect on children’s reading ability. (3) The extra cognitive processing of the TOJ task can significantly affect ATS and has a predictive effect on children’s reading ability.

## 2. Methods

### 2.1. Participants

This study was approved by the ethics committee of East China Normal University, and written informed consent was obtained from the children, their parents, and the adult participants.

The participants included 53 CD, 53 age- and grade-matched TD children, and 37 adults (AD) without a history of dyslexia or other neurodevelopmental disabilities. All participants were native Putonghua speakers, and their eyesight or corrected eyesight and hearing were normal.

The children were recruited from two primary schools. They were from 8 to 12 years old and in grades 3–5. The CD were selected based on the following inclusion and exclusion criteria (Xiong and Yan, 2014; Chen et al., 2016; Zhang et al., 2018): (1) Their level of Chinese character recognition was at or below −1.5 standard deviations in their grade; (2) their percentage ranking of nonverbal IQ was above 25%; (3) their reading fluency was below the grade level average; and (4) their performance in the Chinese language course (two important exams at the end of the last semester and the middle of the current semester) was lower than the percentage ranking of 10% in their class. The age- and grade-matched TD control group also had to meet the following criteria: (1) Their level of Chinese character recognition was at or above the grade level average; (2) their nonverbal IQ was matched to that of CD; (3) their reading fluency was at or above the grade level average; and (4) their performance in the Chinese language course was at or above the percentage ranking of 10% in their class. Children with sensory impairments were excluded. To reduce the impact of teaching and other environmental factors on reading ability, CD and TD children were matched from the same class in the same school.

The adult participants consisted of undergraduate and graduate students recruited from two public universities in China. According to their self-reports, none of these participants had a history of dyslexia or other neurodevelopmental disorders. Their vision or corrected vision and hearing were normal. Two of the adult participants completed the TOJ task only due to their personal schedules. The demographic distribution of participants is shown in Table 1.

**Table 1:** Participants’ demographic information and test scores for screening.

Variable	Children with dyslexia (*n* = 53)	Typically developing children (*n* = 53)	Adults (*n* = 37)
*Gender*			
Male, *n* (%)	32 (60.38)	33 (62.26)	8 (21.6)
Female, *n* (%)	21 (39.62)	20 (37.74)	29 (78.4)
Age, mean (*SD*)	9.99 (0.91)	9.97 (1.02)	23.05 (3.87)
*Grade*			
Grade 3, *n* (%)	17 (32.08)	18 (33.96)	NA
Grade 4, *n* (%)	18 (33.96)	17 (32.08)	NA
Grade 5, *n* (%)	18 (33.96)	18 (33.96)	NA
Raven, mean (*SD*)	104.13 (10.08)	103.17 (10.23)	NA
Character recognition, mean (*SD*)	947.56 (240.58)	1794.58 (209.35)	NA
Reading fluency, mean (*SD*)	144.47 (39.55)	206.94 (43.29)	NA
Phonological awareness, mean (*SD*)	28.31 (7.30)	33.42 (5.60)	NA
Orthographic knowledge, mean (*SD*)	72.62 (3.63)	73.74 (3.23)	NA
Rapid naming, mean (*SD*)	45.67 (12.63)	36.47 (6.46)	NA

### 2.2. Behavioral measures

Behavioral measures were used to screen the children participants with or without dyslexia.

#### 2.2.1. Character recognition test

The test items for each grade consist of 210 Chinese characters divided into 10 groups arranged from *easy* to *difficult*. The participants were required to write a word containing the Chinese character that had been given. The score of the group items was the number of correct responses the children gave to each group of items, multiplied by the group’s weighting coefficient. Each group’s scores were added to estimate the number of characters recognized (Wang and Tao, 1993).

#### 2.2.2. Combined Raven’s test

This test comprises the Raven’s Colored Progressive Matrices (Raven, 1941) and the three units of C, D, and E in the Raven’s Standard Progressive Matrices (Raven, 1983). In this study, the test was employed to examine the children’s nonverbal intelligence. The children’s standard scores were calculated according to the norms of Chinese cities (Wang et al., 2007).

#### 2.2.3. Reading fluency test

This test involves a short essay of 510 words. All Chinese characters in the essay should be mastered by children in grade 3 or higher. The children were asked to read the essay aloud quickly and accurately. Their reading time was recorded. The number of words the child read correctly was divided by the time it took to read the essay to yield the number of words read per minute, which is the child’s reading fluency (Bai, 2017).

#### 2.2.4. Phonological awareness test

This test includes four subtests: recognition of final sounds, recognition of initial sounds, tone recognition, and phoneme deletion (Bai, 2017). Each subtest consists of 10 items for a total of 40 items. For the subtests measuring recognition of final sounds, recognition of initial sounds, and tone recognition, the participants had to choose an option (from among three choices) that was different from the target phoneme. For the phoneme deletion subtest, the participants had to write a new Chinese character corresponding to the remaining phonemes after deleting one of the phonemes of a given word, regardless of tone. The examiner presented the items orally, and the participants answered in writing. The score of the phonological awareness test is the sum of the correct items of each subtest.

#### 2.2.5. Orthographic knowledge test

The “Hong Kong Reading and Writing Learning Difficulty Test” (Ho et al., 2000), as revised by Wang (2016), was used to examine Mandarin-speaking children’s orthographic knowledge of Chinese characters. It includes two subtests: (1) judgments of normal or reversed characters and (2) judgments of true or false characters. The first subtest includes 40 common Chinese characters, of which 20 are left–right reversed. The second subtest also encompasses 40 items, of which 20 are true characters and 20 are non-characters. All true characters are idiosyncratic with a very low frequency of use, while non-characters violate the rules of orthography. The test was administered one-on-one and took about 10 min to complete. The score of the orthographic knowledge test is the sum of the correct items of the two subtests.

#### 2.2.6. The rapid automatic naming test

This test includes two subtests: number and color naming. The number naming consists of five different numbers arranged in eight different sequences of combinations. Each combination is arranged in a row, a total of eight rows. Color naming entails using rectangular blocks of four different colors; the size of each color block is 2 cm × 1 cm. The four colors are arranged in random order, but blocks of the same color are not arranged adjacently. There are five blocks of color in each row, for a total of six rows. The children were asked to state the number or color of each block as quickly and accurately as possible from left to right. The time it took to report all numbers or blocks of color was recorded. The rapid automatic naming test was administered one-on-one. The children could usually complete the rapid automatic naming test in 5 min. The score of the rapid automatic naming test is the sum of the time spent in the number naming and color naming subtests.

### 2.3. Experimental stimulus and procedures

#### 2.3.1. Stimulus

The visual stimulus was a black ring with an outer diameter of 2.5 cm, an inner diameter of 1.9 cm, and a resolution of 105 × 105 pixels. A 14-inch laptop with a screen refresh rate of 60 Hz and a resolution of 1920 × 1,080 pixels was used for testing. The visual stimuli were presented on a white background. According to the work of Boenke et al. (2009), different stimulus durations during audiovisual temporal tasks do not have significant impacts on average PSS values; however, a short stimulus duration will increase the variation of PSS values between individuals. Compared with the longer stimulus duration, the attentional imbalance between individuals for visual and auditory modality can be more clearly reflected by the short stimulus duration. The ability to “grab the attention” in the two sensory modalities is an important factor affecting the performance of temporal sensitivity tasks; therefore, the stimulus duration was set to 16.7 ms – as short as possible, considering one screen refresh rate (60 Hz) cycle. A 1,000 Hz pure tone with the same duration of auditory stimulus was generated using Adobe Audit CC software. The sampling frequency was 44,100 Hz. The auditory stimulus was given with the intensity of 70 dB SPL into the sound field. Two computer speakers were located on both sides below the computer monitor. There was no time difference and intensity difference between the ears.

#### 2.3.2. The audiovisual SJ task

An audiovisual stimulus pair composed of the abovementioned visual and auditory stimuli were presented at each trial. The inter-stimulus intervals (ISIs) of the visual and auditory stimuli were set at eight conditions: 25, 40, 63, 100, 158, 251, 398, and 500 ms. There were 40 trials under each ISI condition, including 20 trials in the AV and 20 VA sequences. A total of 320 trials were conducted. The stimulus pairs of various intervals and orders were presented randomly. The participants had to judge whether auditory and visual stimuli appeared synchronously or not by pressing keys: *1* for *synchronous* or *2* for *asynchronous*. The experiment was divided into eight blocks, and each block was composed of 40 trials. The participants were allowed to pause and rest between each block.

The experiment was performed in a bright room with background noise lower than 40 dB (A). E-prime 2.0 was employed to compile and operate the experimental program. The participants sat at a vertical distance of 50 cm from the computer monitor, and their eyes were at the same height as the center of the computer monitor. The visual angle was 2.5°. At the beginning of the experiment, a 1 cm × 1 cm “+” sign, a shaped red fixation point, was presented in the center of the computer monitor to remind the participants of the beginning of the experiment and the position where the visual stimulus would be presented. After an interval of 800–1,000 ms, visual and auditory stimuli quickly appeared, one after another. The participants had to respond quickly and accurately. The next trial was presented if a response was not given within 5 s. Before the formal experiment, eight practice trials were performed in advance. Two synchronous trials and six asynchronous trials were included in practice trials to avoid response bias. Feedback on the responses was given only on practice trials. When the participants had mastered the response method, they could begin the formal experiment. It took about 20 min to complete this experiment.

#### 2.3.3. The audiovisual TOJ task

The TOJ task was only different in the response method from the SJ task. The participants had to judge which mode of stimulation (visual or auditory) appeared first by pressing keys. They had to press *1* for graphics (visual) and *2* for sound (auditory). Of the eight practical trials that were performed in advance, VA and AV trials accounted for 50%, respectively. Feedback was also only given in practice trials. When the participants could not distinguish the order of the stimuli being presented, guessing was encouraged in the formal experiment. The other settings are exactly the same as in the SJ task.

### 2.4. Data analysis

First, we described the participants’ demographic information and compared the scores of screening tests between the two groups.

Second, the percentage of the synchronous responses was used as the dependent variable, and the groups (CD, TD, and AD) and ISIs (the eight different ISI conditions) were employed as independent variables. A repeated-measures analysis of variance (ANOVA) was utilized to compare differences in ATS between children with and without dyslexia and adults in the SJ task.

**Figure 1:**
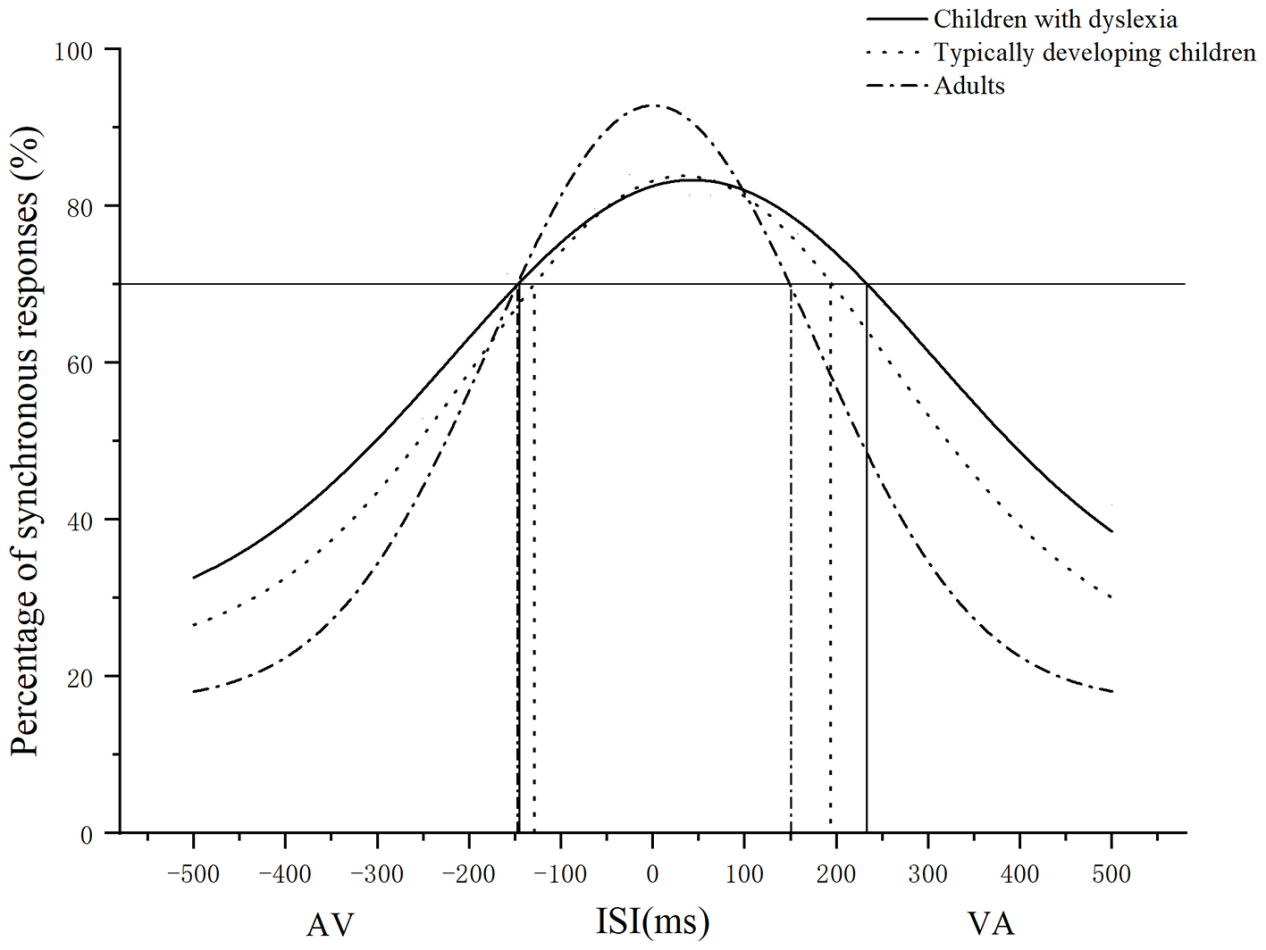
Examples of Gaussian curves fitting the data of three participants in different groups. The ISI value corresponding to the intersection of the horizontal line and the Gaussian curve is the size of the AV (the width between the left vertical line and 0 ms) and VA TBW (the width between 0 ms and right vertical line) when the percentage of synchronous responses is 70%.

Third, to compare the sizes of the TBW between the participants in the different groups, the ISIs were set as the horizontal axis (negative values indicate the AV sequences, while positive values refer to the VA sequences), and the percentage of the synchronous responses was set as the vertical axis (Francisco et al., 2017a). Gaussian fitting was performed for the data of each participant, using MATLAB (Math Works, Inc., Natick, MA). Figure 1 contains the fitting curves for one representative participant in each group. Taking 70% (Stevenson and Wallace, 2013) of the synchronous responses as the critical value, the ISIs corresponding to the critical values in the AV and VA sequences were calculated according to the fitting function. The two ISI values were, respectively, the sizes of the participants’ audiovisual TBW of AV (AV TBW) and VA (VA TBW). The total size of TBW was the sum of the two above. The PSS was the midpoint of the TBW (a negative value indicates the AV direction, a positive value the VA direction). An ANOVA was used to compare the differences in the sizes of the AV TBW, VA TBW, TBW, and the PSS between groups. The reading fluency scores and character recognition were used as the dependent variables. With the demographic variables, the scores of a literacy skills test that correlated significantly with dependent variables, and the sizes of the audiovisual TBW as independent variables, multiple hierarchical regression was performed to examine the predictive effect of the TBW on reading.

Lastly, an analysis of covariance and multiple hierarchical regression was used to analyze the effect of extra cognitive processing in the TOJ task on ATS and reading ability in the condition of controlling the percentage of the synchronous responses of the SJ task.

## 3. Results

Table 1 presents the demographic information of all participants, as well as a comparison of the scores of the screening tests, which were administered to the children with and without dyslexia. There was no significant difference between the two groups in demographic variables (gender: *χ*^2^*_(1, n = 106)_* = 0.04, *p* = 0.842; age: *t _(104)_* = 0.11, *p* = 0.916; grade: *χ*^2^*_(1, n = 106)_* = 0.06, *p* = 0.972; Raven: *t _(104)_* = 0.49, *p* = 0.627) and the score of the orthographic knowledge test (*t _(104)_* = 1.67, *p* = 0.098). TD children performed significantly better than CD in terms of character recognition (*t _(104)_* = 22.17, *p* < 0.001), reading fluency (*t _(104)_* = 7.78, *p* < 0.001), phonological awareness (*t _(104)_* = 3.90, *p* < 0.001), and rapid naming (*t _(104)_* = −4.72, *p* < 0.001) tests.

### 3.1. The development of ATS in children

#### 3.1.1. The percentage of synchronous responses

In the SJ task, the results of ANOVA for the percentage of synchronous responses showed a significant effect of ISI: *F _(7, 966)_* = 259.15, *p* < 0.001, *η*^2^ = 0.653 (VA); *F _(7, 966)_* = 537.55, *p* < 0.001, *η*^2^ = 0.796 (AV) and groups × ISI interaction: *F _(14, 966)_* = 9.44, *p* < 0.001, *η*^2^ = 0.120 (VA); *F _(14, 966)_* = 14.70, *p* < 0.001, *η*^2^ = 0.176 (AV), but no significant effect of groups: *F _(2, 138)_* = 0.92, *p* = 0.402, *η*^2^ = 0.013 (VA); *F _(2, 138)_* = 0.32, *p* = 0.728, *η*^2^ = 0.005(AV). A follow-up test on the interaction indicated that the pattern of groups’ effects was different at specific ISI for VA and AV conditions. VA: ISI = 25 ms, the AD had a significantly larger percentage of synchronous responses than CD and TD children (AD vs. CD, *p* < 0.001; AD vs. TD, *p* = 0.003). ISI = 40 and 63 ms, the percentage of synchronous responses of AD and TD are significant or marginally significantly larger than CD (ISI = 40 ms: AD vs. CD, *p* < 0.001; TD vs. CD, *p* = 0.022; ISI = 63 ms: AD vs. CD, *p* = 0.023 and TD vs. CD, *p* = 0.053). ISI = 100 and 158 ms, there were no significant differences between the three groups (ISI = 100 ms: *F_(2,138)_* = 0.47, *p* = 0.626, *η*^2^ = 0.01; ISI = 158 ms: *F_(2,138)_* = 1.91, *p* = 0.152, *η*^2^ = 0.03). ISI = 251 ms, TD > AD (*p* = 0.013) and CD > AD (*p* = 0.001). ISI = 398 ms, only CD > AD (*p* = 0.002). ISI = 500 ms, CD > TD (*p* = 0.040), CD > AD (*p* < 0.001), and TD > AD (*p* = 0.026). AV: ISI ≤ 100 ms, AD > TD and CD (ISI = 25 ms: AD vs. TD, *p* = 0.011, AD vs. CD, *p* < 0.001; ISI = 40 ms: AD vs. TD, *p* = 0.001, AD vs. CD, *p* < 0.001; ISI = 63 ms: AD vs. TD, *p* < 0.001, AD vs. CD, *p* < 0.001; ISI = 100 ms, AD vs. TD, *p* = 0.002, AD vs. CD, *p* = 0.002). ISI = 158 ms, there were no significant differences between the three groups, *F _(2,138)_* = 1.16, *p* = 0.317, *η*^2^ = 0.02. ISI = 251 ms, CD and TD > AD (CD vs. AD, *p* < 0.001; TD vs. AD, *p* = 0.049). ISI = 398 and 500 ms, CD and TD > AD (ISI = 398 ms: CD vs. AD, *p* < 0.001, TD vs. AD, *p* = 0.008; ISI = 500 ms: CD vs. AD, *p* < 0.001, TD vs. AD, *p* = 0.001), and CD > TD (ISI = 389 ms: CD vs. TD, *p* = 0.021; ISI = 500 ms: CD vs. TD, *p* = 0.007).

#### 3.1.2. The sizes of audiovisual TBW

Gaussian fitting was performed on the percentage of synchronous responses from each participant with changes in ISI. In the groups of CD, TD, and AD, there were eight (15.09%), four (7.5%), and three (8.57%) pieces of data, respectively, that could not be fitted. Table 2 depicts the mean sizes of the VA TBW, AV TBW, TBW, and the mean values of the PSS of the remaining participants in the three groups.

**Table 2:** The sizes of the audiovisual TBW and PSS values.

	VA TBW	AV TBW	TBW	PSS
Children with dyslexia (*n =* 45)	241.28 (125.50)	153.01 (84.78)	394.29 (181.31)	44.13 (57.02)
Typically developing children (*n =* 49)	185.55 (114.50)	128.68 (89.40)	316.34 (169.97)	28.43 (58.11)
Adults (*n =* 32)	163.83 (105.47)	154.28 (54.50)	318.12 (138.12)	4.78 (47.73)

VA TBW = time binding window in VA; AV TBW = time binding window in AV; TBW = total time binding window (AV TBW + VA TBW); PSS = point of subjective simultaneity.

The results of the ANOVA indicated a significant difference in the sizes of TBW and VA TBW between the groups: *F*_(2, 123)_ = 3.10, *p* = 0.049, *η*^2^ = 0.05 (TBW); *F*_(2, 123)_ = 4.75, *p* = 0.010, *η*^2^ = 0.072 (VA TBW). The least significant difference test for multiple comparisons revealed that CD was significantly or marginally significantly larger than TD (TBW: *p* = 0.025; VA TBW: *p* = 0.022) and AD (TBW: *p* = 0.051; VA TBW: *p* = 0.005). However, the difference in the sizes of AV TBW between the three groups was not significant: *F _(2, 123)_* = 1.44, *p* > 0.241, *η*^2^ = 0.02. In addition, the difference in PSS values among the three groups was significant: *F _(2, 123)_* = 4.74, *p* = 0.01, *η*^2^ = 0.072. The outcomes of the least significant difference test for multiple comparisons showed that CD and TD were significantly or marginally significantly larger than AD (CD vs. AD, *p* = 0.003; TD vs. AD, *p* = 0.062). The sizes of the AV TBW and VA TBW within each group were compared. This revealed that the sizes of the VA TBW were highly significantly wider than that of the AV TBW in the two groups of children: *F _(1, 44)_* = 26.96, *p* < 0.001, *η*^2^ = 0.38 (CD); *F _(1, 48)_* = 11.73, *p* = 0.001, *η*^2^ = 0.20 (TD). However, there was no significant difference between the VA TBW and AV TBW within the AD group: *F _(1, 31)_* = 0.32, *p* = 0.575, *η*^2^ = 0.01. When analyzing the data that failed to fit, we found that the data were high for one of the eight CD, three of the TD children, and three AD participants, due to the percentage of synchronous responses from each ISI condition in the VA. Regarding the remaining eight children whose data failed to fit (seven CD and one TD), this was because the percentage of synchronous responses from all ISI conditions was at the chance or lower level whether considering VA or AV, or because the variation range of synchronous response percentage is too small with the change of ISI. Hence, their TBW could not be detected within 500 ms ISI.

#### 3.1.3. The predictive effect of TBW on dyslexia

Multiple hierarchical regression analyses showed that, under the conditions of controlling for the children’s gender, age, and IQ, as well as phonological awareness and rapid naming skills, the size of the VA TBW had a marginally significant predictive effect on children’s reading fluency (Table 3). Neither VA TBW, AV TBW, nor TBW had a significant predictive effect on character recognition.

**Table 3:** Hierarchical regression of TBW on children’s reading fluency.

Hierarchy	Variables	*β*	*t*	*R* ^2^	Δ*R*^2^	*p*
1	Gender	0.01	0.05	0.01	0.01	0.889
	Age	0.09	0.73			
	Raven	0.07	0.61			
2	Phonological awareness	0.29	2.98	0.37	0.36	<0.001
	Rapid naming	−0.43	−4.80			
3	VA TBW	−0.17	−2.00	0.39	0.03	0.060

VA TBW, time binding window in VA.

### 3.2. The effect of extra cognitive processing in the TOJ task on children’s reading ability

The analysis of covariance demonstrated that after controlling for the percentage of synchronous responses in the SJ task, there were still significant differences in the percentage of error responses among the three groups of participants in the TOJ task: *F _(2, 137)_* = 14.65, *p* < 0.001, *η*^2^ = 0.18 (VA); *F _(2, 137)_* = 4.39, *p* = 0.014, η^2^ = 0.06 (AV). The results of the least significant difference test for multiple comparisons showed that CD > TD (*p* = 0.016) and AD (*p* < 0.001), and TD > AD (*p* = 0.001) in the VA condition. In the AV condition, CD and TD > AD (CD vs. AD, *p* = 0.004; TD vs. AD, *p* = 0.028). This suggests that there were also significant differences among the three groups of participants in terms of extra cognitive processing during the TOJ task, after controlling for simultaneity perception abilities. Given the significant differences between the CD and TD group only in the VA condition, a multiple hierarchical regression was employed to analyze the predictive effect of extra cognitive processing in the TOJ task in the VA condition on children’s reading ability, after controlling for the percentage of synchronous responses. However, there was no significantly predictive effect on children’s reading fluency, nor on their character recognition.

## 4. Discussion

This study compared the performance of children with or without dyslexia and normal adults on the audiovisual SJ and TOJ tasks. For the first time, we described in detail the differences in the sizes of the TBW and PSS for simple nonverbal audiovisual stimuli among Chinese children with and without dyslexia and adults, and analyzed the predictive effect of the size of the TBW on children’s reading ability.

### 4.1. The ATS of 8–12-year-old children is immature

In the SJ task, when the ISI was small, the percentage of synchronous responses of the adults was significantly higher than that of both groups of children. Simultaneous processing for auditory and visual stimuli that are very close in time could help individuals to obtain redundant information from multimodal stimuli. With increasing ISI, the percentage of synchronous responses of adults showed a more rapid decline than that of both groups of children. In particular, when the ISI was greater than their threshold of synchronous perception, the adults could more explicitly perceive two stimuli as occurring asynchronously than the children. The discrimination of asynchronous stimuli with longer ISI facilitates the processing of the stream of audiovisual stimuli, so that the correspondence between visual and auditory stimuli presented in rapid succession is not blurred (Hahn et al., 2014; Wallace and Stevenson, 2014).

The current study found that, although the size of the TBW of Chinese TD children was not significantly different from that of adults, their VA TBW was significantly wider than their AV TBW. Their PSS were also biased toward the VA end, signifying a marked asymmetry. Compared to the children, the TBW of the adults was more symmetrical, and their PSS was closer to the objective simultaneity point. Primarily, the results indicate that even the ATS of the 8–12-year-old TD children was still immature. This outcome differs from that of Hillock et al. (2011), but is consistent with the electrophysiological findings of Froyen et al. (2008, 2009) in adults and children.

There was also no significant difference in the size of the AV TBW between children and adults. This suggests that there are different trajectories for the development of temporal sensitivity in AV and VA sequences. Contrary to the findings of Kaganovich (2016), the development of ATS in the VA sequences was slower than that in the AV sequences for Chinese children. An analysis of the data that failed to fit also validates this developmental trajectory. According to these data, the VA TBW failed to be detected in the adults and most TD children, but neither the VA TBW nor AV TBW could be detected in most of the CD group. The VA TBW developed more slowly than the AV TBW. A TD child who was unable to detect VA and AV TBW showed an irregular synchronous response percentage lower than the guess probability in all ISI conditions; this may be due to the child’s response bias.

### 4.2. The ATS of Chinese children with dyslexia lags behind that of TD children

In both the VA and AV sequences, the ATS of CD lagged behind that of TD children. When the ISI was longer, the CD group had a significantly higher percentage of synchronous responses than the TD children. Their TBW, especially the VA TBW, was wider than that of the TD children. This outcome is similar to the findings of Francisco et al. (2017a) in adults. They found that adults with dyslexia who were native speakers of Dutch lagged more in the VA sequences than in the AV sequences, compared to normal controls, on both the verbal and nonverbal AV SJ tasks. Sound and light travel at different speeds in the air, so visual stimuli often reach the sense organs before simultaneous auditory stimuli. However, the human brain activates auditory stimuli 30–50 ms faster than visual stimuli that are simultaneously received. Thus, to a certain extent, the time gap between perceived stimuli is narrowed and even reversed for visual and simultaneous auditory stimuli presented closer to the person (Arrighi et al., 2006). That is, auditory stimuli are perceived before visual stimuli. Hence, individuals typically have a wider TBW when visual stimuli are presented before auditory stimuli (Van Wassenhove et al., 2007). The results of the current study also support this view. Studies of unimodal temporal sensitivity in Chinese children have found that the visual temporal sensitivity of CD developed more slowly than their auditory temporal sensitivity. Their visual temporal sensitivity reached a level similar to that of TD children of the same age in higher grades (Chung et al., 2008; Wang and Yang, 2018). Poor visual temporal sensitivity may affect the speed of processing of visual stimuli in audiovisual cross-modal processing. Visual stimuli need to appear at longer time intervals before auditory stimuli are presented so that the two modalities of stimulation can be processed to occur asynchronously. That is, poor visual temporal sensitivity may result in a wider TBW for audiovisual integration in the VA sequences. The sequence of decoding of reading happens to follow the character–speech VA sequences. The findings of the regression analysis also showed that the size of the VA TBW had a marginally significant predictive effect on children’s reading fluency. Meanwhile, the size of the AV TBW did not. This suggests that audiovisual VA TBW that is too wide may be one reason for decoding deficits found in people with dyslexia. However, further empirical investigation is needed to confirm this inference.

Furthermore, the orthographic knowledge of CD group did not have significant defects. It is consistent with the findings of Chung et al. (2008) and Xu et al. (2001). The orthographic knowledge test was used to recognize the static character structure in this study. In contrast, the defect of ATS has a greater impact on text reading that requires continuous and dynamic processing of different characters, such as reading fluency. It is suggested that the defects of Chinese dyslexia are related to the difficulty in deploying attention resources effectively in the process of character conversion, so that it cannot accurately and quickly correspond to character–speech (Stein, 2019). The obstacle to character structure recognition is not the main factor of dyslexia.

### 4.3. The effect of extra cognitive processing in the TOJ task on children’s ATS

In this study, the TOJ task only asked participants to report which modality of stimulus came first, without reporting on the stimulus’s characteristics. Compared with the audiovisual TOJ task in the work of Liu et al. (2019), the TOJ task reduced the participants’ cognitive load. Although after controlling for the performance of SJ task, the extra cognitive processing in the TOJ task did not significantly affect reading ability in the current study, it was still significant for affecting participant’s ATS as the view of Skottun and Skoyles (2010). Especially in the VA sequences, the CD exhibited stronger extra cognitive processing effects than the TD children. Dyslexia is often co-morbid with defects in higher cognitive processing functions, such as working memory and attention (Gabay et al., 2020; Tadi and Aghaei, 2021). Audiovisual processing tasks with higher levels of cognitive load require the involvement of other cognitive processing abilities. Hence, the study’s findings would be more influenced by each individual’s higher cognitive function. This may confound the effects of participants’ other cognitive functions and audiovisual time perception ability in relation to the experimental results.

## 5. Limitations

In this study, the lack of literate age-matched TD children compared to CD made it impossible to determine whether the deficits in ATS in CD are developmental or specific. In addition, as no reading ability screening test suitable for adults was available, the adult participants were only identified as having no history of dyslexia through self-report. Students enrolled in public universities in China need to pass strict examinations, including many reading tests for Chinese language courses. Adults with dyslexia can hardly pass the entrance examination. However, the lack of objective assessment of adult reading ability will still reduce the interpretability of the results.

## 6. Conclusion

First, the ATS of 8 to 12-year-old Chinese TD children in this study was not yet mature. Second, the ATS of children with dyslexia lagged behind that of their TD peers significantly, and their VA TBW was wider than that of TD children. The width of the VA TBW had a marginally significant predictive effect on children’s reading ability. Third, compared to the SJ task with the same stimulus material and ISI, the extra cognitive processing in the TOJ task had a significant effect on the children’s ATS, but no significant predictive effect on their reading ability.

## Data availability statement

The raw data supporting the conclusions of this article will be made available by the authors, without undue reservation.

## Ethics statement

The studies involving human participants were reviewed and approved by the ethics committee of East China Normal University. Written informed consent to participate in this study was provided by the participants’ legal guardian/next of kin.

## Author contributions

QL and HW conceived the original idea. HW and HL planned and carried out the experiment. HW and QL analyzed the experimental data. HW wrote the manuscript with support from QL and YZ. All authors contributed to the article and approved the submitted version.

## Funding

The Educational Sciences Planning Project of Zhejiang Province (grant number: 2022SCG384).

## Conflict of interest

The authors declare that the research was conducted in the absence of any commercial or financial relationships that could be construed as a potential conflict of interest.

## Publisher’s note

All claims expressed in this article are solely those of the authors and do not necessarily represent those of their affiliated organizations, or those of the publisher, the editors and the reviewers. Any product that may be evaluated in this article, or claim that may be made by its manufacturer, is not guaranteed or endorsed by the publisher.

## Abbreviations

ATS: audiovisual temporal sensitivity
TBW: time binding window
TOJ: temporal order judgment
SJ: simultaneity judgment
PSS: point of subjective simultaneity
CD: children with dyslexia
TD: typically developing
AD: adults
VA: visual stimuli preceding auditory stimuli
AV: auditory stimuli preceding visual stimuli
ISI: inter-stimulus intervals

## References

[ref1] Arrighi R. Alais D. Burr D. (2006). Perceptual synchrony of audiovisual streams for natural and artificial motion sequences. J. Vis. 6, 6–268. doi: 10.1167/6.3.6, PMID: 16643094

[ref2] Bai L. (2017). A New Approach to the Assessment and Subtype Diagnosis of Developmental Dyslexia. Tianjin: Nankai University Press. 371–372.

[ref3] Binder M. (2015). Neural correlates of audiovisual temporal processing: comparison of temporal order and simultaneity judgments. Neuroscience 300, 432–447. doi: 10.1016/j.neuroscience.2015.05.011, PMID: 25982561

[ref4] Birch H. G. Belmont L. (1964). Auditory visual integration in normal and retarded readers. Am. J. Orthopsychiatry 34, 852–861. doi: 10.1111/j.1939-0025.1964.tb02240.x, PMID: 14220514

[ref5] Boenke L. T. Deliano M. Ohl F. W. (2009). Stimulus duration influences perceived simultaneity in audiovisual temporal-order judgment. Exp. Brain Res. 198, 233–244. doi: 10.1007/s00221-009-1917-z, PMID: 19590862

[ref6] Chen L. Zhang M. Ai F. Xie W. Meng X. (2016). Crossmodal synesthetic congruency improves visual timing in dyslexic children. Res. Dev. Disabil. 55, 14–26. doi: 10.1016/j.ridd.2016.03.010, PMID: 27022720

[ref7] Chung K. K. McBride-Chang C. Wong S. W. Cheung H. Penney T. B. Ho C. S. (2008). The role of visual and auditory temporal processing for Chinese children with developmental dyslexia. Ann. Dyslexia 58, 15–35. doi: 10.1007/s11881-008-0015-4, PMID: 18483866

[ref8] Francisco A. A. Groen M. A. Jesse A. McQueen J. M. (2017b). Beyond the usual cognitive suspects: the importance of speechreading and audiovisual temporal sensitivity in reading ability. Learn. Individ. Differ. 54, 60–72. doi: 10.1016/j.lindif.2017.01.003

[ref9] Francisco A. A. Jesse A. Groen M. A. McQueen J. M. (2017a). A general audiovisual temporal processing deficit in adult readers with dyslexia. J. Speech Lang. Hear. Res. 60, 144–158. doi: 10.1044/2016_JSLHR-H-15-0375, PMID: 28056152

[ref10] Franzen L. Stark Z. Johnson A. P. (2021). Individuals with dyslexia use a different visual sampling strategy to read text. Sci. Rep. 11:6449. doi: 10.1038/s41598-021-84945-9, PMID: 33742007 PMC7979812

[ref11] Froyen D. J. W. Bonte M. L. Van Atteveldt N. Blomert L. (2009). The long road to automation: neurocognitive development of letter-speech sound processing. J. Cogn. Neurosci. 21, 567–580. doi: 10.1162/jocn.2009.21061, PMID: 18593266

[ref12] Froyen D. J. W. Van Atteveldt N. V. Bonte M. Blomert L. (2008). Cross-modal enhancement of the MMN to speech-sounds indicates early and automatic integration of letters and speech-sounds. Neurosci. Lett. 430, 23–28. doi: 10.1016/j.neulet.2007.10.014, PMID: 18023979

[ref13] Gabay Y. Gabay S. Schiff R. Henik A. (2020). Visual and auditory interference control of attention in developmental dyslexia. J. Int. Neuropsychol. Soc. 26, 407–417. doi: 10.1017/S135561771900122X, PMID: 32238215

[ref14] Hahn N. Foxe J. J. Molholm S. (2014). Impairments of multisensory integration and cross-sensory learning as pathways to dyslexia. Neurosci. Biobehav. Rev. 47, 384–392. doi: 10.1016/j.neubiorev.2014.09.007, PMID: 25265514 PMC4258132

[ref15] Hairston W. D. Burdette J. H. Flowers D. L. Wood F. B. Wallace M. T. (2005). Altered temporal profile of visual-auditory multisensory interactions in dyslexia. Exp. Brain Res. 166, 474–480. doi: 10.1007/s00221-005-2387-6, PMID: 16028030

[ref16] Hillock A. R. Powers A. R. Wallace M. T. (2011). Binding of sights and sounds: age-related changes in multisensory temporal processing. Neuropsychologia 49, 461–467. doi: 10.1016/j.neuropsychologia.2010.11.041, PMID: 21134385 PMC3140703

[ref17] Hillock-Dunn A. Wallace M. T. (2012). Developmental changes in the multisensory temporal binding window persist into adolescence. Dev. Sci. 15, 688–696. doi: 10.1111/j.1467-7687.2012.01171.x, PMID: 22925516 PMC4013750

[ref18] Ho C. S. H. Chan D. W. O. Tsang S. M. Lee S. H. (2000). The Hong Kong Test of Specific Learning Difficulties in Reading and Writing (HKT-SpLD) Manual. Hong Kong: Hong Kong Specific Learning Difficulties Research Team.

[ref19] Jaśkowski P. (1991). Two-stage model for order discrimination. Percept. Psychophys. 50, 76–82. doi: 10.3758/bf03212206 1881768

[ref20] Kaganovich N. (2016). Development of sensitivity to audiovisual temporal asynchrony during miCDhildhood. Dev. Psychol. 52, 232–241. doi: 10.1037/dev0000073, PMID: 26569563 PMC4821764

[ref21] Kaganovich N. Schumaker J. Leonard L. B. Gustafson D. Macias D. (2014). Children with a history of SLI show reduced sensitivity to audiovisual temporal asynchrony: an ERP study. J. Speech Lang. Hear. Res. 57, 1480–1502. doi: 10.1044/2014_JSLHR-L-13-0192, PMID: 24686922 PMC4266431

[ref22] Laasonen M. Tomma-Halme J. Lahti-Nuuttila P. Service E. Virsu V. (2000). Rate of information segregation in developmentally dyslexic children. Brain Lang. 75, 66–81. doi: 10.1006/brln.2000.2326, PMID: 11023639

[ref23] Lewkowicz D. J. (1996). Perception of auditory-visual temporal synchrony in human infants. J. Exp. Psychol. Hum. Percept. Perform. 22, 1094–1106. doi: 10.1037//0096-1523.22.5.1094, PMID: 8865617

[ref24] Lewkowicz D. J. Flom R. (2014). The audiovisual temporal binding window narrows in early childhood. Child Dev. 85, 685–694. doi: 10.1111/cdev.12142, PMID: 23888869 PMC3954953

[ref25] Liu S. Wang L. C. Liu D. (2019). Auditory, visual, and cross-modal temporal processing skills among Chinese children with developmental dyslexia. J. Learn. Disabil. 52, 431–441. doi: 10.1177/0022219419863766, PMID: 31313628

[ref26] Love S. A. Petrini K. Pernet C. R. Latinus M. Pollick F. E. (2018). Overlapping but divergent neural correlates underpinning audiovisual synchrony and temporal order judgments. Front. Hum. Neurosci. 12:274. doi: 10.3389/fnhum.2018.00274, PMID: 30018545 PMC6037859

[ref27] Lyon G. R. Shaywitz S. E. Shaywitz B. A. A. (2003). A definition of dyslexia. Ann Dyslexia 53, 1–14. doi: 10.1007/s11881-003-0001-9

[ref28] Matsuzaki K. S. Kadota H. Aoyama T. Takeuchi S. Sekiguchi H. Kochiyama T.. (2014). Distinction between neural correlates of audiovisual temporal order and simultaneity judgments. Int. J. Psychophysiol. 94:193. doi: 10.1016/j.ijpsycho.2014.08.801

[ref29] Mégevand P. Molholm S. Nayak A. Foxe J. J. (2013). Recalibration of the multisensory temporal window of integration results from changing task demands. PLoS One 8:e71608. doi: 10.1371/journal.pone.0071608, PMID: 23951203 PMC3738519

[ref30] Naples A. J. Chang J. T. Katz L. Grigorenko E. L. (2009). Same or different? Insights into the etiology of phonological awareness and rapid naming. Biol. Psychol. 80, 226–239. doi: 10.1016/j.biopsycho.2008.10.002, PMID: 19007845 PMC2708917

[ref31] Raven J. C. (1941). Standardization of progressive matrices, 1938. Br. J. Med. Psychol. 19, 137–150. doi: 10.1111/j.2044-8341.1941.tb00316.x

[ref32] Raven J. (1983). “The progressive matrices and Mill Hill vocabulary scale in western societies” in Human Assessment and Cultural Factors. eds. Irvine S. H. Berry J. W. (New York: Springer), 107–114.

[ref33] Skottun B. C. Skoyles J. R. (2010). Temporal order judgment in dyslexia: task difficulty or temporal processing deficiency? Neuropsychologia 48, 2226–2229. doi: 10.1016/j.neuropsychologia.2010.04.013 20412813

[ref34] Stein J. (2019). The current status of the magnocellular theory of developmental dyslexia. Neuropsychologia 130, 66–77. doi: 10.1016/j.neuropsychologia.2018.03.022, PMID: 29588226

[ref35] Stevenson R. A. Wallace M. T. (2013). Multisensory temporal integration: task and stimulus dependencies. Exp. Brain Res. 227, 249–261. doi: 10.1007/s00221-013-3507-3, PMID: 23604624 PMC3711231

[ref36] Tadi V. T. Aghaei A. (2021). Comparison of relationship between the working memory and false memory in dyslexia and normal students. J. Except. Child. 21, 103–114. Available at https://www.researchgate.net/publication/352478431

[ref37] Tallal P. Piercy M. (1973). Defects of non-verbal auditory perception in children with developmental aphasia. Nature 241, 468–469. doi: 10.1038/241468a0, PMID: 4705758

[ref38] Van Eijk R. L. Kohlrausch A. Juola J. F. van de Par S. (2008). Audiovisual synchrony and temporal order judgments: effects of experimental method and stimulus type. Percept. Psychophys. 70, 955–968. doi: 10.3758/pp.70.6.955, PMID: 18717383

[ref39] Van Wassenhove V. V. Grant K. W. Poeppel D. (2007). Temporal window of integration in auditory-visual speech perception. Neuropsychologia 45, 598–607. doi: 10.1016/j.neuropsychologia.2006.01.001, PMID: 16530232

[ref40] Vatakis A. Navarra J. Soto-Faraco S. Spence C. (2008). Audiovisual temporal adaptation of speech: temporal order versus simultaneity judgments. Exp. Brain Res. 185, 521–529. doi: 10.1007/s00221-007-1168-9, PMID: 17962929

[ref41] Virsu V. Lahti-Nuuttila P. Laasonen M. (2003). Crossmodal temporal processing acuity impairment aggravates with age in developmental dyslexia. Neurosci. Lett. 336, 151–154. doi: 10.1016/s0304-3940(02)01253-3, PMID: 12505615

[ref42] Wallace M. T. Stevenson R. A. (2014). The construct of the multisensory temporal binding window and its dysregulation in developmental disabilities. Neuropsychologia 64, 105–123. doi: 10.1016/j.neuropsychologia.2014.08.005, PMID: 25128432 PMC4326640

[ref43] Wang X. (2016). Chinese Developmental Dyslexia and PASS Cognitive Processing. Beijing: China Social Sciences Press, 127–128.

[ref44] Wang D. Di M. Qian M. (2007). A report on the third revision of the combined Raven’s test (CRT-C3) for children in China. Chin. J. Clin. Psychol. 15, 559–568. Available at https://wenku.baidu.com/view/fcaf08e8ecfdc8d376eeaeaad1f34693dbef1036?fr=xueshu_top&_wkts_=1680686273166

[ref45] Wang X. Tao B. (1993). Chinese Character Recognition Test Battery and Assessment Scale for Primary School Children. Shanghai: Educational Press.

[ref46] Wang L.-C. Yang H.-M. (2018). Temporal processing development in Chinese primary school–aged children with dyslexia. J. Learn. Disabil. 51, 302–312. doi: 10.1177/0022219416680798, PMID: 27940605

[ref47] Xiong J. Yan G. (2014). Analysis of the main subtypes of Chinese developmental dyslexia. Stud. Psychol. Behav. 12, 496–500. Available at https://wenku.baidu.com/view/8150dabaa3116c175f0e7cd184254b35eefd1a90?fr=xueshu_top&_wkts_=1680686539774

[ref48] Xu S. Peng D. Tan L. (2001). The mental mechanism of developmental dyslexia in children: a preliminary study. Psychol. Dev. Educ. 17, 12–16, 22. doi: 10.3969/j.issn.1001-4918.2001.04.003

[ref49] Zampini M. Shore D. I. Spence C. (2003). Audiovisual temporal order judgments. Exp. Brain Res. 152, 198–210. doi: 10.1007/s00221-003-1536-z 12879178

[ref50] Zhang M. Xie W. Xu Y. Meng X. (2018). Auditory temporal perceptual learning and transfer in Chinese-speaking children with developmental dyslexia. Res. Dev. Disabil. 74, 146–159. doi: 10.1016/j.ridd.2018.01.005, PMID: 29413429

